# Influence of Oxidation Damages on Mechanical Properties of SiC/SiC Composite Using Domestic Hi-Nicalon Type SiC Fibers

**DOI:** 10.1155/2020/8840963

**Published:** 2020-12-08

**Authors:** Enze Jin, Denghao Ma, Zeshuai Yuan, Wenting Sun, Hao Wang, Jiajia Zhang, Xiaodong Gong, Lina Huang, Botao Han, Xin Sun, Zhihai Feng, Junping Li

**Affiliations:** Key Laboratory of Advanced Functional Composites Technology, Aerospace Research Institute of Materials & Processing Technology, Beijing 100076, China

## Abstract

Here, we show that when the oxidation treatment temperature exceeded 600°C, the tensile strength of SiC/SiC begins to decrease. Oxidation leads to the damages on the PyC fiber/matrix interface, which is replaced by SiO_2_ at higher temperature. The fracture mode converts from fiber pull-out to fiber-break as the fiber/matrix interface is filled with SiO_2_. Oxidation time also plays an important role in affecting the tensile strength of SiC/SiC. The tensile modulus decreases with temperature from RT to 800°C, then increases above 800°C due to the decomposition of remaining CSi_*x*_O_*y*_ and crystallization of the SiC matrix. A special surface densification treatment performed in this study is confirmed to be an effective approach to reduce the oxidation damages and improve the tensile strength of SiC/SiC after oxidation.

## 1. Introduction

The SiC/SiC composite is known to have high specific strength, high specific stiffness, high temperature resistance, long-term oxidation resistance, and erosion resistance [1–3], all of which are ideal properties for application as high-temperature-resistant material in many fields such as thermal protection system of aerospace vehicles and hot-end components of aircraft engines [1–3]. In addition, the SiC/SiC composite reinforced by a near-stoichiometric SiC fiber is considered the candidate material for the new-generation fuel claddings of nuclear fission reactor and the first wall material of nuclear fusion reactor due to its high irradiation resistance [4, 5].

The performance of the SiC/SiC composite oxidized in air is very important in the engineering application of this material, which draws widely interests in recent years [6, 7]. Lu et al. investigated the oxidation damages of SiC/SiC composites with pyrolytic carbon (PyC) fiber/matrix interface using high-resolution CT and SEM. The results showed that PyC interface was severely oxidized and turns into voids above 1000°C. After oxidation at 1400°C, the brittle oxide layers filled cracks in the matrix and caused drop of tensile strength [8]. Ikarashi et al. studied the tensile properties of 3D woven SiC/SiC composites at high temperatures in air. They found that oxidative matrix crack propagation in the transverse layers strongly affected the lifetime of the SiC/SiC composites [9]. Wang et al. studied the tensile creep properties of two-dimensional (2D) woven SiC/SiC composites reinforced with low-oxygen and high-carbon SiC fibers at high temperature. The results revealed that the bridged fiber inhibited the opening of matrix cracks; on the other hand, the fiber creep promoted the growth of crack. The creep property was determined by the compete mechanisms of fibers at high temperature [10].

Domestic Hi-Nicalon type SiC fiber is a new material, and there are few studies on the oxidation damages of SiC/SiC composites prepared by this fiber [11, 12]. In this study, we will investigate the influence of oxidation damages on mechanical properties of the SiC/SiC composite reinforced by domestic Hi-Nicalon type SiC fibers under high-temperature environments.

## 2. Materials and Methods

2D preforms were woven using domestic Hi-Nicalon type SiC fibers. The SiC fibers were provided by Xiamen University (Xiamen, China). The fiber diameter is 14 *μ*m, density is 2.79 g/cm, tensile strength is 2.7 GPa, and the modulus is 270 GPa. The oxygen content is 0.5 wt%, and the C/Si mole ratio is 1.41. The properties of the domestic Hi-Nicalon type SiC fiber are close to those of Hi-Nicalon fiber [13, 14]. PyC layers with a thickness of about 400 nm were coated on the surface of the preforms by chemical vapor deposition (CVD) method. SiC/SiC composites were prepared by precursor infiltration and pyrolysis (PIP) process. The preforms were impregnated with liquid-state polycarbosilane (PCS) by a vacuum infiltration method and pyrolyzed at 800°C in an inert argon atmosphere. The impregnation and pyrolysis process were repeated 10 times until weight increase was less than 1%. Five specimens were fabricated from the composite to have an accurate calculation of density and porosity considering the Archimedes method based on ASTM C373-88 standard. The averaged porosity of the composite is 8.1%.

The machined SiC/SiC composite mechanical specimens were subjected to oxidation treatment in a muffle furnace with a temperature increasing rate of 100°C/min until the required temperature was achieved. The tensile properties of the materials were tested at room temperature using a servoelectric testing system (MTS CMT5105, China). The length, width, and height of tensile specimen are 100 mm, 5 mm, and 10 mm, respectively. Five specimens were tested for each state, and the strength was obtained by averaging the values over these five results.

## 3. Results and Discussion

### 3.1. Mechanical Property Variation

**Figure 1:**
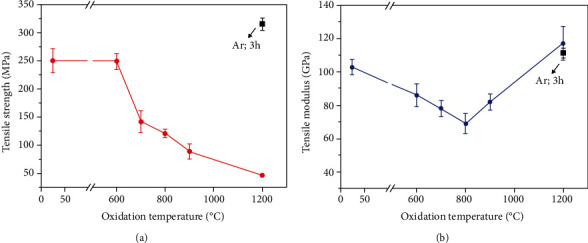
Variations of (a) tensile strength and (b) tensile modulus of SiC/SiC oxidized in air at different temperatures.

The tensile strength used in this study is the maximum stress that the SiC/SiC composite can withstand while being stretched before fracture. Young's modulus is obtained from the linear portion of the stress-strain curve in the SiC/SiC composite. The variation of tensile strength of SiC/SiC composites after oxidation for 3 hours at 600°C, 700°C, 800°C, 900°C, and 1200°C is shown in Figure 1(a). It can be seen from the results that the tensile strength of SiC/SiC composites decreases with oxidation temperature during 600-1200°C. However, the tensile strength of SiC/SiC composites treated at 1200°C under inert gas environment is slightly higher than that of as-fabricated specimens. It indicates that the reduction of tensile strength is mainly due to the oxidation damages. The tensile strength of SiC/SiC composites barely change after oxidation at 600°C for 3 hours. When the oxidation temperature exceeds 600°C, the tensile property begins to drop. After oxidation at 800°C for 3 hours, the retention of tensile strength is ~50%, and after oxidation at 1200°C, the retention of tensile strength is only ~20%.

The variations of tensile modulus of SiC/SiC composites after oxidation at different temperatures are summarized in Figure 1(b). The change of tensile modulus with temperature is different with that of tensile strength. The tensile modulus decreases with temperature from 600°C to 800°C, and then, the tensile modulus increases with oxidation temperature until 1200°C.

The results of tensile property variations reveal that the oxidation damages of the SiC/SiC composite occur after 3 hours of oxidation at 600°C. The oxidation damages at 600°C only have influences on the tensile modulus of the material and have almost no influence on the tensile strength. When the oxidation temperature exceeds 600°C, the tensile strength of SiC/SiC composites begins to decline. The tensile modulus starts to increase above 800°C, which is also the preparation temperature of the SiC/SiC composite. When the temperature exceeds 800°C, the remaining CSi_*x*_O_*y*_ component in the matrix was further converted into SiC and then crystallized with the increase of temperature [15]. The modulus of the CSi_*x*_O_*y*_ fiber (Nicalon fiber) is much lower than that of SiC fiber (Hi-Nicalon fiber) [14]. Therefore, it is reasonable to infer that the increase of modulus above 800°C is caused by the conversion of the remaining CSi_*x*_O_*y*_ component in the matrix.

**Figure 2:**
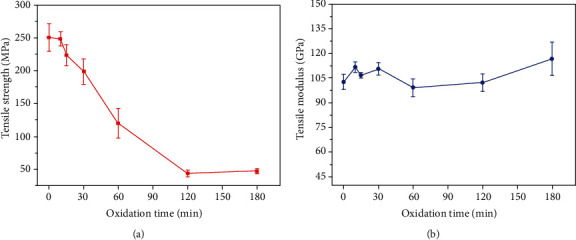
Variations of (a) tensile strength and (b) tensile modulus of SiC/SiC oxidized in air at 1200°C for different times.

The change of tensile strength of SiC/SiC composites after oxidation at 1200°C for 10 min, 15 min, 30 min, 60 min, 120 min, and 180 min is shown in Figure 2(a). It can be seen from the results that oxidation time also plays an important role to affect the tensile strength of SiC/SiC composites. SiC/SiC composite strength begins to decrease when the oxidation time exceeds 10 min. When the oxidation time reaches 120 min, the tensile strength does not further decrease.

The variation of tensile modulus of SiC/SiC composites oxidized at 1200°C as a function of time is shown in Figure 2(b). It can be seen from the results that the effect of oxidation time on the tensile modulus of SiC/SiC is not readily observable.

Both oxidation time and temperature can affect the tensile strength of SiC/SiC composites. In this study, we use an oxidation damage parameter, *q*, to consider the influence of temperature and time comprehensively.

(1) $$\begin{array}{c} q=T\log\left(t\right), \end{array}$$

where *T* is oxidation temperature and *t* is oxidation time. We define *r* as the tensile strength loss rate,

(2) $$\begin{array}{c} r=\frac{\left(\sigma_{\text{As}}-\sigma_{\text{Ox}}\right)}{\sigma_{\text{As}}}\times 100\%, \end{array}$$

where *σ*_As_ is the tensile strength of as-received SiC/SiC, *σ*_Ox_ is the tensile strength of SiC/SiC after oxidization treatment. We use oxidation damage parameter, *q*, to establish the prediction model of tensile strength loss rate, *r*:

(3) $$\begin{array}{c} r=\left\{\begin{array}{cc} 0 & \left(i\text{f}q\leq 0\right),\\ aT\log\left(t\right)+b & \left(\text{if}q>0\right). \end{array}\right. \end{array}$$

**Figure 3:**
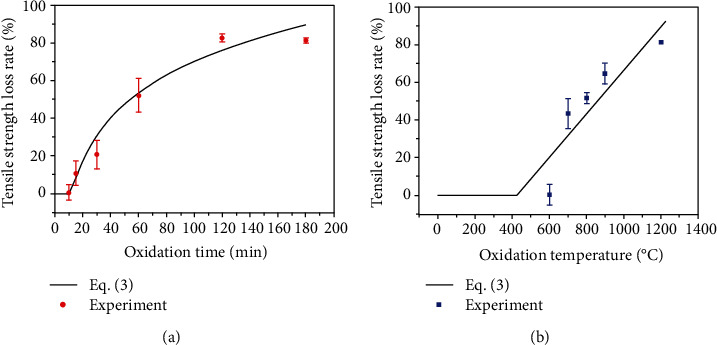
(a) Variations of tensile strength loss rate at 1200°C for different oxidized time and (b) tensile strength loss rate oxidized at different temperature for 3 hours.

We can get *a* = 0.051 and *b* = −80.41 by data fitting. The relationship between tensile strength loss rate and time oxidized at 1200°C is shown in Figure 3(a). The relationship between tensile strength loss rate and oxidation temperature (3 hours of oxidation) is shown in Figure 3(b). It can be seen that the predictive curve of SiC/SiC composite tensile strength loss rate obtained based on oxidation damage parameter is in good agreement with the experimental results.

### 3.2. Microstructures

**Figure 4:**
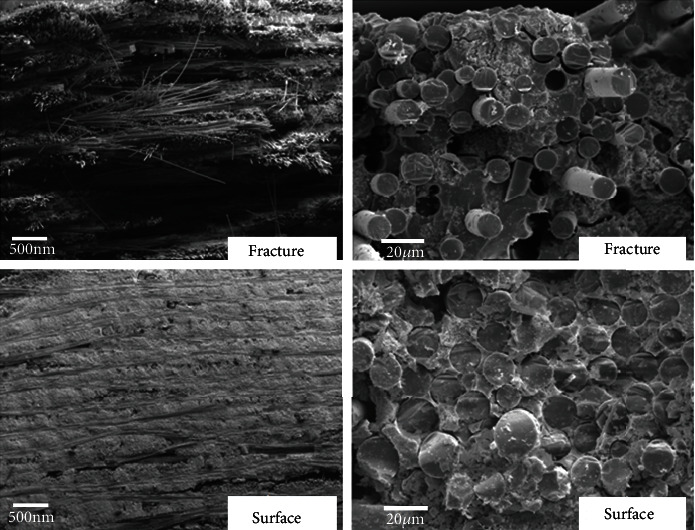
Tensile fracture morphology of SiC/SiC composites oxidized in air at 600°C for 3 hours.

The microstructures at the fracture of SiC/SiC composites were characterized by Camscan Apollo 300 scanning electron microscope (CamScan, Cambridge, UK). The failure mode and microstructure damages can be investigated in the fractures of specimens [13, 14]. The microstructures of SiC/SiC composite oxidized for 3 hours at 600°C are shown in Figure 4. It can be seen that the fractures of the SiC/SiC composite at 600°C exhibit ductile characteristics. A large number of extracted fibers can be clearly seen at the fracture, indicating that the crack was deflected at the PyC fiber/matrix interface during the loading process. The results show that the PyC fiber/matrix interface on the surface of the specimen has disappeared, while the PyC interface at the fracture still exists. It indicates that the oxidation damages at 600°C only occurred on the surface of the material. Oxygen atoms do not further oxidized the PyC interface layer inside the material.

**Figure 5:**
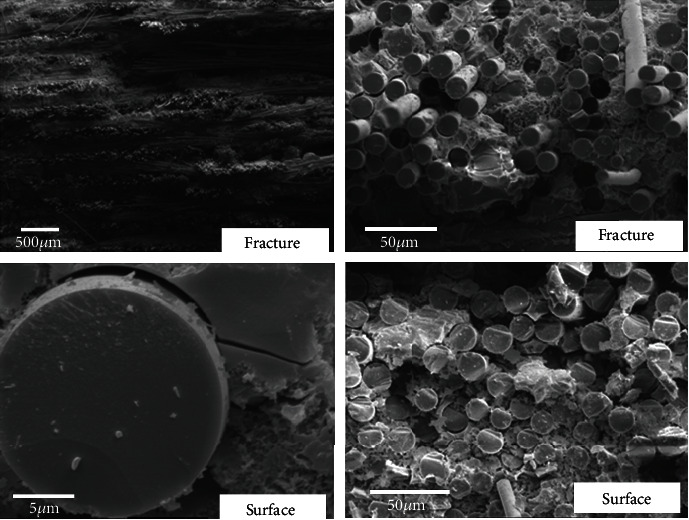
Tensile fracture morphology of SiC/SiC composites oxidized in air at 800°C for 3 hours.

The microstructures of SiC/SiC composite specimens oxidized for 3 hours at 800°C are shown in Figure 5. Compared with the composite oxidized at 600°C, the length of pulling out fibers decreases and the regions of pulling out fibers become smaller. The results show that no PyC interface at the fracture can be seen after oxidation at 800°C for 3 hours. Therefore, oxidation damage occurred inside the composite under this condition, and nearly all of the PyC interfaces have been oxidized. When the PyC at the fiber/matrix interface is oxidized, pores are formed between the fiber and matrix. Pulled-out fibers can also be seen at the fractures, indicating that cracks are deflected during the loading process. SiC/SiC composite oxidized at 800°C for 3 hours still exhibits ductile fracture behavior. However, due to the loss of the interface layer, loading stress cannot transfer effectively between the fiber and matrix. Therefore, the strength and modulus of SiC/SiC composites drop significantly on this condition. This result suggests that the rupture under constant tensile load is caused mainly by the oxidation of SiC interphace because of air ingression through the transverse crack.

**Figure 6:**
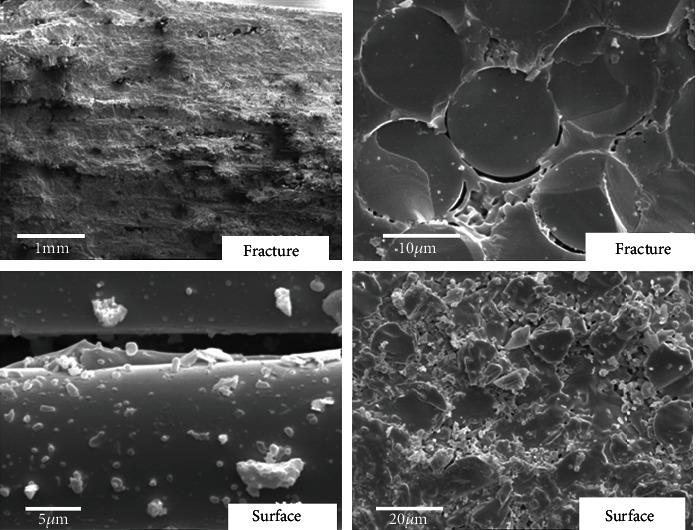
Tensile fracture morphology of SiC/SiC composites oxidized in air at 1200°C for 3 hours.

The microstructures of SiC/SiC composites oxidized for 3 hours at 1200°C are shown in Figure 6. The fracture surface of the composites is very plane, and few pullout fibers can be seen. The fractures show typical brittle characteristic. It indicates that cracks are not arrested at the interface but penetrated the fiber bundles. Further observation reveals that the pores caused by the oxidation of PyC are sealed at the fiber/matrix interface. It is because the oxidations of SiC matrix and the fiber at 1200°C: SiC+O_2_→SiO_2_+CO_2_+CO. SiO_2_ filled the pores between the fiber and matrix becomes a new fiber/matrix interface. Pores between the fiber and matrix which are not filled completely are also shown in Figure 6. Oxidation of PyC interface results in strong chemical bonding between fibers and the matrix. During the tensile process, crack initiations in SiC/SiC composites mainly appear in the matrix and gradually propagate to the fiber/matrix interface. If the fiber/matrix interface is weak enough, the crack will deflect into the interface and propagate along fiber axis direction. If the interface is too strong, the crack will not deflect but directly cause fiber break [16, 17]. Interphase usually plays a key role in determining the mechanical properties of materials [18–20]. In our previous work, both theory and experiment show that once the interface binding strength exceeds the critical value, the composite failure mode converts from fiber pull-out ductile failure mode to fiber break brittle failure mode and the tensile strength of SiC/SiC drops sharply [21]. It is worthy to mention that because sufficient SiO_2_ filled the pores on the material surface, oxygen atoms prevent further entry into the material [22, 23]. It can explain the fact that the tensile strength did not further decrease with time when oxidized at 1200°C above 120 min as shown in Figure 2(a).

**Figure 7:**
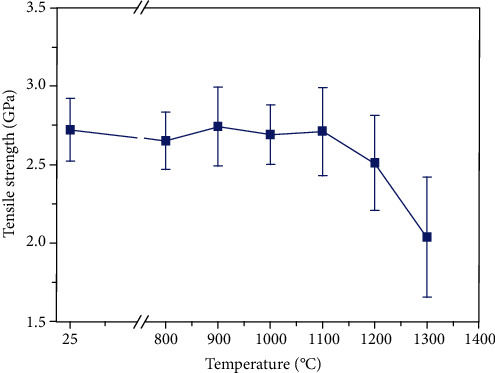
Variations of SiC fiber oxidized for 3 h in air at different temperatures.

According to the theory of composite mechanics, the tensile strength of the SiC fiber also plays a key role in the mechanical properties of SiC/SiC. We performed the oxidation treatment on domestic Hi-Nicalon type SiC fibers at different temperatures using the same method as composite oxidation treatment. Then, the fiber tensile strength was measured by Instron tensile device using a load cell of 5 N and a crosshead speed 4.5 mm/min. The gauge length is 25 mm, and at least 50 samples were tested to calculate the average tensile strength. The variations of tensile strength of domestic Hi-Nicalon type SiC fibers with oxidation temperature are shown in Figure 7. It can be seen that the tensile strength of the SiC fiber begins to decrease above 1100°C. SiC fibers still retain high strength after oxidized at 1200°C and retention rate is 92%. These results indicate that the drop of the SiC/SiC composite oxidized from RT to 1200°C is mainly caused by the oxidation of PyC interphase, rather than the oxidation of the SiC fiber.

### 3.3. Improvement

**Figure 8:**
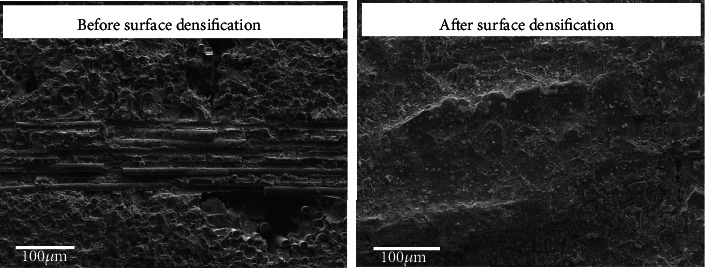
Comparison of surface morphology of SiC/SiC before and after surface densification treatment.

The micropores of SiC/SiC composites act as a pathway of oxygen, which are mainly caused by the shrinkage during PCS pyrolysis and machining process. In order to increase the densification of the surface and prevent the oxygen diffuse into the SiC/SiC composite, we prepared mixed precursor with 60 wt% SiC powders (Shanghai ST-Nano Co. Ltd., China, purity: 99%, grain size < 100 nm) and 40 wt% PCS to reduce the precursor shrinkage during pyrolysis. The SiC-50 wt% PCS powders were homogeneously mixed by ball milling at 250 r/min for 8 h to obtain uniformly flowing ceramic slurry, in which xylene was selected as solvent. One additional impregnation and pyrolysis process was performed using the mixed precursor to seal the micropores on the surface of as-fabricated specimens. The results show that no micropore can be seen on the surface of the composite after additional impregnation and pyrolysis process (as shown in Figure 8).

**Figure 9:**
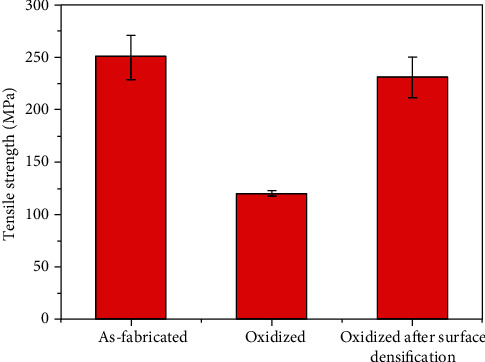
Comparison of tensile strength of SiC/SiC before and after surface densification treatment (oxidized at 1200°C for 1 hour).

The SiC/SiC composites with densified surface were oxidized for 1 hour at 1200°C. The comparison between tensile strength of SiC/SiC with and without densified surface is shown in Figure 9. From the results, it can be seen that the tensile strength of SiC/SiC composites after surface densification is significantly improved, which is close to the strength before oxidation treatment. It indicates that the densified surface prevent the oxygen diffuse into the SiC/SiC composite and reduce the oxidation damages effectively.

**Figure 10:**
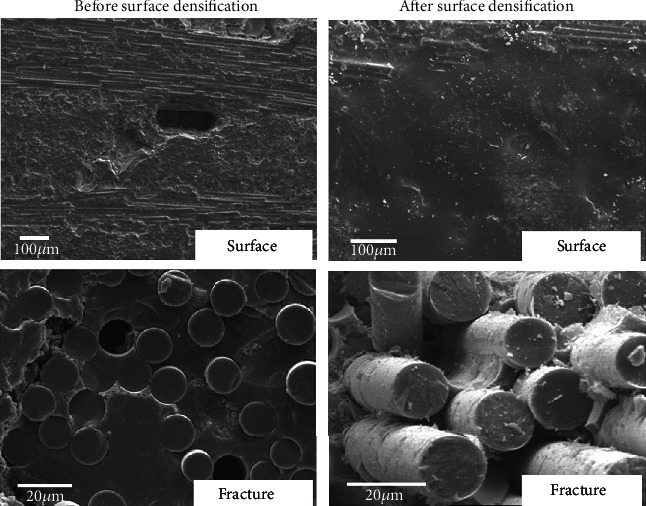
Comparison of tensile fracture morphology of SiC/SiC before and after surface densification treatment (oxidized at 1200°C for 1 hour).

The microstructures of oxidized SiC/SiC composites before and after surface densification are shown in Figure 10. It can be seen from the figure that the surface of the as-fabricated composite has obvious pores. The PyC interface is replaced by SiO_2_ between the fibers and matrix in the fracture. The composites exhibit brittle characteristics. The surface of the SiC/SiC composite is covered by a dense SiO_2_ film after surface densification, which effectively prevents oxygen diffusion into the composite. PyC can be seen in the fracture of the SiC/SiC composite, indicating that no oxidation damages occur inside the composite. Due to the PyC interface remaining intact, the interface strength between the fiber and matrix is low, leading to ductile fracture and improved tensile strength after oxidation.

## 4. Conclusions

The tensile properties of SiC/SiC reinforced with domestic Hi-Nicalon type SiC fibers oxidized at high temperature were investigated in this study. Both oxidation temperature and time have significant effects on the tensile properties of SiC/SiC. The tensile property begins to decrease with temperature when the oxidation temperature exceeds 600°C. The drop of tensile strength is mainly caused by the replacement of the PyC fiber/matrix interface by SiO_2_ during oxidation. The tensile modulus decreases with temperature from RT to 800°C, then increases above 800°C due to the decompose of the remaining CSi_*x*_O_*y*_ and crystallization of the SiC matrix. A tensile strength loss rate model as a function of oxidation temperature and time is proposed. The prediction roughly agreed with the experimentally obtained results. Surface densification treatment is an effective way to reduce the oxidation damages and improve the tensile strength of SiC/SiC.

## Data Availability

The data can be easily obtained in the figures in manuscript.
